# MUNDUS project: MUltimodal Neuroprosthesis for daily Upper limb Support

**DOI:** 10.1186/1743-0003-10-66

**Published:** 2013-07-03

**Authors:** Alessandra Pedrocchi, Simona Ferrante, Emilia Ambrosini, Marta Gandolla, Claudia Casellato, Thomas Schauer, Christian Klauer, Javier Pascual, Carmen Vidaurre, Margit Gföhler, Werner Reichenfelser, Jakob Karner, Silvestro Micera, Andrea Crema, Franco Molteni, Mauro Rossini, Giovanna Palumbo, Eleonora Guanziroli, Andreas Jedlitschka, Marco Hack, Maria Bulgheroni, Enrico d’Amico, Peter Schenk, Sven Zwicker, Alexander Duschau-Wicke, Justinas Miseikis, Lina Graber, Giancarlo Ferrigno

**Affiliations:** 1 NeuroEngineering And medical Robotics Laboratory, NearLab, Department of Electronics, Information, and Bioengineering, Politecnico di Milano, Italy; 2 Control Systems Group, Technische Universität Berlin, Berlin, Germany; 3 Machine Learning Group, Computer Science Faculty, Technische Universität Berlin, Berlin, Germany; 4 Technische Universität Wien, Vienna, Austria; 5 Translational Neural Engineering Lab, Center for Neuroprosthetics, Ecole Polytechnique Federale de Lausanne (EPFL), Lausanne, Switzerland; 6 The BioRobotics Institute, Scuola Superiore Sant’Anna, Pisa, Italy; 7 Valduce Hospital, Villa Beretta Rehabilitation Center, Costa Masnaga, Lecco, Italy; 8 Fraunhofer Institute for Experimental Software Engineering, Kaiserslautern, Germany; 9 Ab.Acus, Milan, Italy; 10 Hocoma AG, Volketswil, Switzerland

**Keywords:** Assistive device, Upper limb support, Neuromuscular electrical stimulation, Wearable exoskeleton, Neurological disorders

## Abstract

**Background:**

MUNDUS is an assistive framework for recovering direct interaction capability of severely motor impaired people based on arm reaching and hand functions. It aims at achieving personalization, modularity and maximization of the user’s direct involvement in assistive systems. To this, MUNDUS exploits any residual control of the end-user and can be adapted to the level of severity or to the progression of the disease allowing the user to voluntarily interact with the environment. MUNDUS target pathologies are high-level spinal cord injury (SCI) and neurodegenerative and genetic neuromuscular diseases, such as amyotrophic lateral sclerosis, Friedreich ataxia, and multiple sclerosis (MS). The system can be alternatively driven by residual voluntary muscular activation, head/eye motion, and brain signals. MUNDUS modularly combines an antigravity lightweight and non-cumbersome exoskeleton, closed-loop controlled Neuromuscular Electrical Stimulation for arm and hand motion, and potentially a motorized hand orthosis, for grasping interactive objects.

**Methods:**

The definition of the requirements and of the interaction tasks were designed by a focus group with experts and a questionnaire with 36 potential end-users.

Five end-users (3 SCI and 2 MS) tested the system in the configuration suitable to their specific level of impairment. They performed two exemplary tasks: reaching different points in the working volume and drinking. Three experts evaluated over a 3-level score (from 0, unsuccessful, to 2, completely functional) the execution of each assisted sub-action.

**Results:**

The functionality of all modules has been successfully demonstrated. User’s intention was detected with a 100% success. Averaging all subjects and tasks, the minimum evaluation score obtained was 1.13 ± 0.99 for the release of the handle during the drinking task, whilst all the other sub-actions achieved a mean value above 1.6. All users, but one, subjectively perceived the usefulness of the assistance and could easily control the system. Donning time ranged from 6 to 65 minutes, scaled on the configuration complexity.

**Conclusions:**

The MUNDUS platform provides functional assistance to daily life activities; the modules integration depends on the user’s need, the functionality of the system have been demonstrated for all the possible configurations, and preliminary assessment of usability and acceptance is promising.

## Background

Restoring and augmenting human capabilities compensating for reduced motor functions and disabilities may be carried out by different approaches, all of them finalised to return to the involved person some missing functions or capabilities. The types of functions that are worthwhile to be restored strictly depend on the personal history and life of the entailed subject [1].

The International Classification of Functioning, Disability and Health (ICF) copes well with subjectivity in the identification of the functions able to guarantee human dignity and self-esteem. The recognition of the person, with his/her history, willing and wishes, is a key point in the development of methods to overcome disabilities and augment human capabilities. Human dignity and self-esteem are more preserved when restoring missing functions with devices safeguarding self-perception and first hand interaction while guaranteeing independent living. ICF identifies facilitators and barriers as environmental factors which, through their presence (facilitator) or absence (barrier), improve activity and functions, or reduce disability. Assistive technologies market offers a wide range of facilitators designed to support independent life.

People coming from a personal history of severe traumas or neuromuscular diseases that have led to a sudden or progressive loss of motor capabilities attribute a high value to the maintenance of a direct interaction with daily life objects [2]. Simple tasks, such as taking autonomously a glass, bringing it to the mouth and drinking, are actions that contribute to a positive assessment of their own quality of life. However, most of the assistive technologies solutions for people with severe motor impairments hardly surrogate the natural human interaction with daily life objects [3]. Passive functional upper limb orthoses (e.g. [4,5]) are mainly used for rehabilitation purposes. Power assisted exoskeletons (exo) (e.g. [6,7]) are basically developed for stationary rehabilitation exercising in a clinical environment, and they are rather heavy due to the power-demanding actuators integrated into the system. A different approach, recently investigated in literature, is the use of assistive robotic manipulators which can be mounted to the side of an electric-powered wheelchair for general manipulation [8,9]. However, these solutions work without a continuous control by the user’s intention and are not usually connected to the user’s arm.

In general, robotic arms have not been very successful in the past because of their cumbersomeness, high cost and reduced acceptability by the users, even if some interesting examples have been recently discussed in the literature, such as the upper limb assistive device based on Neuro Muscular Electrical Stimulation (NMES) proposed by Shill and colleagues, which has the primary goal to improve the paralyzed upper extremity function and, thus, to enhance the patient’s independence in activities of daily living [10].

An innovative solution may be offered by customizable and modular systems able to exploit any residual motor capability and assure a direct interaction of the user with the external environment, preserving the most the naturalness. This is the way pursued by the MUNDUS project through the implementation of a new concept of a modular assistive neural prosthesis to support basic arm and hand functions, such as reaching and grasping. The MUNDUS assistive neural prosthesis helps the user to reach an object, by positioning the arm in the space, to grasp it, and to bring it to a target final destination (the mouth or any location of the user’s workspace).

Expected MUNDUS users are people affected by high-level Spinal Cord Injury (SCI) and neurodegenerative diseases such as Amyotrophic Lateral Sclerosis (ALS), Friedreich Ataxia, Multiple Sclerosis (MS). Except for SCI, all of these pathologies are characterized by a progressive course of the impairment with a faster or slower continuous loss of motor capabilities. It is very important to cope with the current motor condition day by day, offering solutions able to be modularly adapted to the current modifying status of the person. In severe neurodegenerative impairments, the possibility to deploy the same assistive device, properly changing its configuration, from the early phase of the disease to the latest one, is a key issue to increase acceptability of the system itself and to enhance its usability.

**Figure 1:**
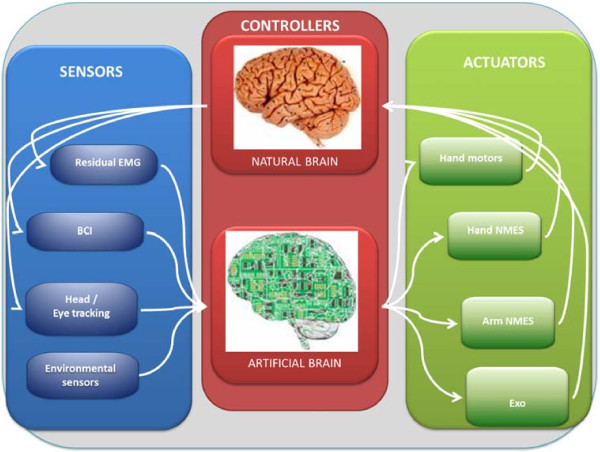
MUNDUS concept.

This approach was adopted in literature in the robotic rehabilitation of the lower limbs, by developing patient-cooperative control strategies able to adapt the robot controller to the patient’s voluntary effort [11-14]. The concept of MUNDUS is to apply a similar approach to assistive devices for upper limb support in order to increase the usability and acceptability of the system by maximizing the user involvement in the task execution. Indeed, MUNDUS offers a modular solution able to follow the user in the progression of the disease: sensors, actuators and control solutions can be adapted to the actual level of severity, allowing interaction through the voluntary control of the user (Figure 1).

On the control level, MUNDUS exploits a modular and expandable set of voluntary commands that the user is able to send. In case of impairment of neuromuscular functions, there are few exploitable commanding strategies to detect the intention to move and “where to go”: electromyography (EMG) signals [15,16], by taking advantage of residual local neuromuscular function; head or eyes motion [17]; and brain signals acquired by electroencephalography (EEG) [18,19], when muscular activities are no more available. MUNDUS pursues the modular implementation of these possible strategies and, based on the user, the therapist/clinician selects the control signals according to his/her residual capabilities.

On the execution level, MUNDUS allows the choice of actuators, again, according to available personal resources. Whenever possible, arm motion is powered by the user’s own muscles, and facilitated by gravity compensation provided by a passive, spring-loaded exoskeleton. Alternatively, NMES is delivered to the upper arm muscles to induce the arm movement within the defined workspace. The use of NMES successfully combines the naturalness of the function, which is still performed by the arm/hand muscles, with some systemic and local benefits. Positive fallouts of the daily use of assistive technologies based on NMES are the increase of muscle tone, the reduction of spasticity, the bone remineralisation, and a training effect of motor relearning over the central nervous system [20-25]. NMES allows the system to be artificially empowered without worsening its wearability and lightness.

At the hand level, when the user is not anymore able to functionally use his/her own hand, an NMES actuated grasping glove or a specifically designed robotic orthosis are available to assist the grasping of collaborative “functional objects”, recognized by Radio Frequency Identification (RFID).

The purpose of the present paper is to provide a global overview of the MUNDUS platform and of its first validation on end-users. In the Methods section, the users and clinical requirements of the whole system are described; the system modules are described and the evaluation methods are outlined. Details on the technical design and the implemented solutions for each module are not reported as well as the validation of the modules on healthy subjects: single publications are referred for these parts. The results are then focused on end-users experiments. A group of 5 subjects affected by neurological diseases tested the system in different configurations according to their current level of disability. To assess the performance, a 3-level score for functional evaluation was autonomously assigned by three experts.

## Methods

### Definition of users and clinical requirements

The MUNDUS system was developed adopting a user-centred approach: the design process started with capturing the clinical requirements through a focus group of experts and interviews of potential users, and continued the development and optimization of the system until all the possible user needs were fulfilled.

A focus group was brought together to identify the possible applications of the MUNDUS platform and to suggest requirements (see Additional file 1 - Focus group and potential user group questionnaires and answers). Fourteen experts were recruited for the focus group: 7 medical doctors (5 physiatrists, 1 neurologist, 1 general medical doctor), 1 psychologist, 1 physiotherapist, 1 mechanical engineer, 1 patient affected by Amyotrophic Lateral Sclerosis, 1 caregiver, and 2 social enterprise representatives employing disabled people. The main required aspects were modularity, reproduction of movements as close as possible to “natural” ones in terms of performance, preference for low encumbering device, multitask device to be used in different tasks/environments, reasonable costs and ease of use.

A group of potential users was also identified. A total of 39 MUNDUS potential users have been contacted and 36 gave their consent to participate to the interview (see Additional file 1 - Focus group and potential user group questionnaires and answers). The analysis of the potential users’ interviews yielded some of the design inputs for the device. The most required activities were related to daily living: eating, drinking, and personal hygiene. The major required goal was to improve autonomy. They would like to have a device useable mostly at home during the activities of daily living. The device should be easy to use, light, and wearable, even if all the selected users depended on a wheelchair.

The data collected from the focus group and the users’ interviews suggested that the device should allow at least the following movements: anterior reaching, hand to mouth, hand to body, antigravity support of arm, and gross grasping (not fine movement of the fingers). Further, the requirement of portability was set as less crucial for the MUNDUS platform because most of end-users depended on a wheelchair; thus, the use of the MUNDUS system was restricted to a table.

### Definition of users scenarios and interaction tasks

Depending on the users’ residual capabilities, three different scenarios have been identified. Specifically, subjects grouped in scenario 1 present residual functional control of the arm and/or hand muscles, but they are too weak to accomplish functional tasks in daily activities. The residual EMG signals of the upper limb muscles is used to drive the system. In scenario 1, the allowed interaction tasks are not pre-defined because they strongly depend on the residual capabilities of the user.

Within scenario 2, subjects have no residual functional voluntary activation of arm and hand muscles, but they can still control the head and gaze fixation. Then, an eye tracking system is used to identify the intention of the users.

Subjects belonging to scenario 3, even if not blind, lack the ability to move their eyes and, thus, they are not able to reliably fix different locations of the screen, which is a prerequisite for using an eye tracking system. The interaction with these subjects is only performed by brain signals, as recorded by EEG.

For scenarios 2 and 3, a set of interaction tasks has been pre-defined to fulfil the clinical requirements. The following tasks have been included: pressing a button, drinking with a straw, eating (even if most of subjects at this level of disability are affected by dysphagia, i.e. dysfunctions in the neural control of swallowing), touching their own body, changing the posture of the other arm, bringing an object to the face (e.g. a towel or a sponge), touching another person, interacting with objects for personal hygiene (e.g. a brush or a wet napkin). To simplify the control of the movement and to optimize the interaction between the user and the system, each interaction task has been divided in sub-actions. For instance, the drinking task has been divided into 6 sub-actions: (1) going from rest to the cup position on the table, (2) grasping the cup, (3) going to the mouth, (4) going back to the table, (5) releasing the cup, and (6) going back to the rest position. The triggering of most of the sub-actions should be given by the user, so as to allow him/her to keep a direct control of the function.

### Description of the system modules

#### Sensors used to detect the user’s intention

The detection of the user’s intention is performed in different ways, depending on the residual capabilities of the user, i.e. depending on the scenarios. The following systems can be alternatively used: an EMG amplifier and/or a USB-button (scenario 1), an eye tracking system (scenario 2), and a Brain Computer Interface (BCI) (scenario 3).

These modules share the following functions: selection of the final target point to be reached at the beginning of each interaction tasks and triggering of specific sub-actions.

##### EMG & USB-button module

In scenario 1, EMG surface electrodes are used to detect the residual activation of the arm muscles with a double aim: to modulate arm NMES in order to augment the volitional muscle contractions of the user, assuring the completion of the task; and to trigger the execution of the sub-actions. A USB-button controlled by the contralateral hand of the user can be used to substitute the detection of the EMG signal for the triggering of the sub-actions [26].

A multi-channel signal amplifier system (Porti™, Twente Medical System International) is used to acquire the EMG signals at 2048 Hz. EMG recordings take place on the shoulder (anterior, medial and posterior deltoid), and on the upper arm (biceps). A user-defined muscle of the contralateral arm is also acquired when the EMG signal is used for triggering the sub-actions. The EMG amplifier and the signal processing shall assure the acquisition of the residual volitional EMG in the presence of stimulation artefacts coming from NMES [26,27].

##### Eye Tracking module

The eye tracker is provided by a commercial device and only specific GUI for the MUNDUS application have been developed. The Tobii T60® system, a table mounted eye tracker integrated into a 17” TFT monitor, has been selected. During tracking, the Tobii T60 uses infrared diodes to generate reflection patterns on the corneas of the user’s eyes. Proper image processing is used to identify the gaze point on the screen. One Kinect camera is used to show on the screen the live scene of the objects on the table the subject can choose to interact with, while special parts of the screen are dedicated to other available tasks (i.e., emergency button, touching spots of the body). To trigger the sub-actions, specific questions are displayed on the screen and the user can reply by fixating a GO or a STOP icon.

##### BCI module

The BCI control is based on the Center Speller [28], but applied to an object selector. The advantage of this interface, based on Event Related Potentials (ERP), is that it can be operated by non-spatial feature attention. By paying attention to a rare event between a sequence of frequent ones, a time and phase locked positive polarity is evoked in the EEG. To infer which action/object the user tries to select, spatio-temporal features of the ERPs are extracted with machine learning techniques and used to feed a Linear Discriminant Classifier [29]. To trigger the sub-actions, specific questions are displayed on the screen and the user can reply by selecting a GO or a STOP icon.

The brain activity is acquired from the scalp with multi-channel EEG BrainAmp amplifiers (Brain Products GmbH) using an ActiCap with 16 Ag/AgCl electrodes in an extended 10–20 system sampled at 1000 Hz with a band-pass filter from 0.05 to 200 Hz.

#### Sensors used to monitor and control the movement

##### HAND sensors

To properly monitor the hand functions, it is important to detect basic hand joint movements and interaction forces with objects [30]. A sensorised glove was designed, manufactured, and assembled. The glove is light, unobtrusive, and highly transpiring. Bend sensors (Bend Sensors, Flexpoint Sensor Systems Inc) on metacarpal and proximal interphalangeal joints were used to assess the kinematic configuration of the hand, while force sensors, placed under the finger tips and on the palm, were used to detect grasp contact points and grasp force (Tekscan A201 and A401 Force Sensing Resistors, FSR).

##### Exoskeleton sensors and environmental sensors

The exoskeleton includes encoders to measure the angles at the three Degrees Of Freedom (DOF) of the arm (Vert-X, Contelec AG, Switzerland).

Environmental sensors are used to identify and track the elements (hand, mouth, and objects) in the working volume, and to provide their absolute 3D positions within a common coordinate system. One Kinect™ sensor is used to identify and track the position of the hand and the objects on the table (top-view camera). Making use of the speed of the exo sensors and the accuracy of the environmental sensors, by means of calibration and filtering, an accuracy of about ±1 cm and data rate of about 50 Hz is achieved.

#### Interactive objects

All the objects are equipped with a RFID tag to make them automatically recognizable, and to activate the correspondent workflow. In this way, among the possible interactive objects selected for scenario 2 and 3, the system automatically acts properly once the arm is approaching the object as soon as the RFID antenna mounted on the exo distal element read the tag. This solution avoids the caregiver to daily inform the system of the used objects, which could be eventually also changed during the session without any rebooting procedure. The selected passive tags are low cost adhesive rectangular tags easily attachable on any support.

The RFID reader used is the R1230CB QUARK by CAENRFID working in the 865.600÷867.600 MHZ range (ETSI EN 302 208).

To allow a safe handling of the objects for the desired interaction tasks, a special handle with cardanic joints has been developed. Different standard objects can be mounted on the handle, with minor adjustments, so to allow the most of the interaction tasks, without the development of specific single objects. The handle can be either used to keep the object verticality, as for the glass, or to fix the object at any other orientation, such as for the brush.

#### Actuators

##### Exoskeleton

The exoskeleton provides 2 DOFs at the shoulder: shoulder elevation in the sagittal plane and shoulder rotation in the horizontal elevation plane. The third DOF at the shoulder (rotation of the homers around its axis) is locked permanently. At the elbow, one DOF is provided.

When the pronation/supination of the forearm or the flexion/extension of the wrist are not under user control, MUNDUS fixes these two DOFs through the mechanical structure. The locking of the wrist rotation as well as that of the humeral rotation can be efficiently compensated by using the designed handle and holder.

**Figure 2:**
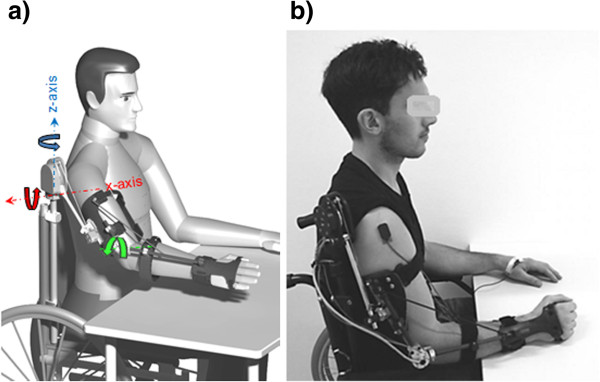
**The exoskeleton. a)** Digital mock-up of the exo-Version 2; **b)** The exo worn by a test participant in a wheelchair.

Two exo prototypes are available, Version 1 for persons with residual motor function who only need weight support, as provided by passive elements (springs and elastic wires), and Version 2 with additional electromagnetic DC brakes for locking of the mechanical DOFs (Kendrion http://www.kendrion.com). The exo - Version 2 has a total weight of 2.2 kg, while Version 1 weighs 1.4 kg. Figure 2 shows a digital mock-up of the exo-Version 2 (panel a) and a test participant sitting in a wheelchair and donning the exo (panel b).

##### ARM NMES controller

An 8-channel current-controlled stimulator (RehaStim™, Hasomed GmbH), delivering rectangular biphasic pulses is used to provide NMES to the arm muscles. The stimulation frequency is constant and fixed at 25 Hz for all stimulation channels, whereas the pulse amplitude and the pulse width range are set individually on each channel. In order to induce arm movements at the shoulder and elbow joints, the following muscles are stimulated with standard adhesive electrodes (PALS® Platinum, Axelgaard Manufacturing Co., Ltd.): the biceps and the anterior, median and posterior deltoid. The triceps is not stimulated because elbow’s extension is assured by gravity.

According to the scenario, the stimulation commands are controlled in two different ways.

In scenario 1, when used, NMES is controlled by the residual EMG volitional activity. The volitional EMG activity is on-line converted into an integral control of the duration of the current pulses delivered to the muscle. Two thresholds set on each user define the level of muscular activation to start and stop the stimulation [26].

In scenario 2/3, a feedback controller is used to induce arm movements by means of NMES. This controller has been designed as a single DOF control sequence exploiting the selective blocking of the other degrees as provided by the exo brakes. The calculation of an angular reference position is achieved by computing the inverse kinematics for a given 3D target position. For the shoulder elevation, a digital controller based on an identified dynamic transfer function model is automatically designed using the pole-placement method in the calibration phase. The control of the horizontal shoulder rotation as well as the elbow-joint angle is achieved by constantly ramping-up the stimulation intensity until the reference angle is reached and locked with the corresponding brake. A sequential feedback controller has been preferred to a simultaneous feedback control of the 3 DOFs integrated with a biomimetic feedforward controller [31] able to mimic the naturalness of the arm movement [32-34]. Indeed, the use of the sequential feedback controller alone can reduce the calibration time and assure a very robust accuracy in reaching the target, which is the most relevant requirement for MUNDUS.

##### HAND NMES controller

**Figure 3:**
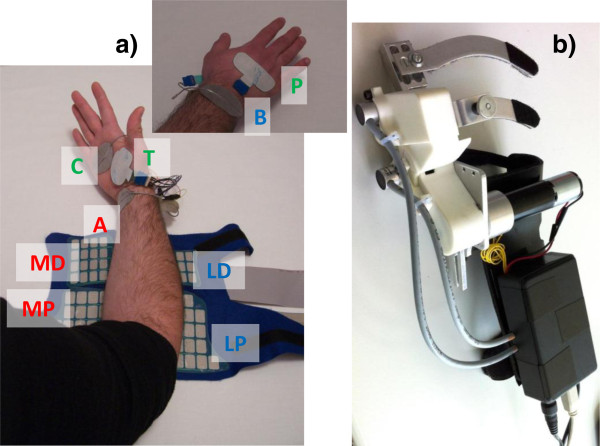
**The hand module. a)** The stimulation arrays embedded in the garment: MD, medial distal, and MP, medial proximal for finger flexion (indifferent electrode A); LD, lateral distal, and LP, lateral proximal for finger extension (indifferent electrode B); T, thenars, and P, palmar (indifferent electrode C). **b)** The robotic hand orthosis.

A second stimulator (RehaStim™, Hasomed GmbH) is used to control NMES of the forearm and hand muscles. Since electrode arrays are used, a customized demultiplexer is connected to the stimulator. At the hand level, NMES induces flexion of the fingers joints to get a palmar grip, and extension of the fingers joints to achieve hand opening movement and consequently to release the object [35,36]. Extrinsic flexors, extrinsic extensors, thenar muscles, and lumbricals, palmar and dorsal interossei muscles are stimulated. The design of the electrode array offers a good trade-off between NMES selectivity and device complexity [37]. Figure 3 (panel a) shows the garment with stimulation arrays embedded for hand NMES.

As shown in Figure 3 (panel a) a total of 6 arrays are used: the medial distal (MD) and medial proximal (MP) arrays are used to stimulate the fingers flexors; the lateral distal (LD) and lateral proximal (LP) arrays for the fingers extensors; and two small electrode arrays are used to stimulate the lumbricals, dorsal and palmar interossei (P) and the thenars muscles (T), respectively. Three indifferent electrodes, indicated as A, B, and C, are used in combination with the electrode arrays. An initial calibration, automatically driven by a dedicated software, is required to check the forearm muscles response to NMES and select which element of each array will be used and to set the stimulation parameters (pulse amplitude and duration) to best assure the completion of each single action. The stimulation frequency is fixed at 20 Hz for all stimulation channels.

The timing of the stimulation of the different muscles is pre-planned [38-40] taking into account the information coming from the interactive object.

##### Robotic hand orthosis

In the case of complete absence of any muscular activity regarding hand motor functions or in the case of hypersensitivity to electrical hand stimuli, an actuated robotic hand orthosis, shown in Figure 3 (panel b) has been designed. This orthosis has two coupled DOFs driven by a DC motor with a planetary gearhead (A-max 22 and GP 22, Maxon motor, Switzerland) and two angular sensors (Vert-X, Contelec AG, Switzerland) to measure the MetaCarpoPhalangeal (MCP) and the Proximal InterPhalangeal (PIP) joint angles. The MCP and PIP joint motions are coupled with a fixed gear ratio through a timing belt transmission. The orthosis, characterized by a total weight of 0.51 kg, can be adjusted to different hand and fingers lengths. The robotic hand orthosis is mechanically mounted to the distal part of the arm allowing for free palmar grasping of cylindrical objects. The thumb is fixed in opposition to the fingers by means of a soft and flexible orthopaedic thumb brace.

#### Mundus central controller and real-time control

The overall control of the modules is set by the MUNDUS Central Controller (MUNDUS CC), a state machine controller communicating with all modules. For the purpose of the system integration, the single module controllers have been integrated into two PCs – one Linux-based computer running the real time controller and one Windows-based computer running MUNDUS CC. The communication between the modules is established via UDP and messages are broadcasted in the XML format. MUNDUS CC as a state machine handles all use cases by reacting upon receiving trigger messages and broadcasting state commands.

**Figure 4:**
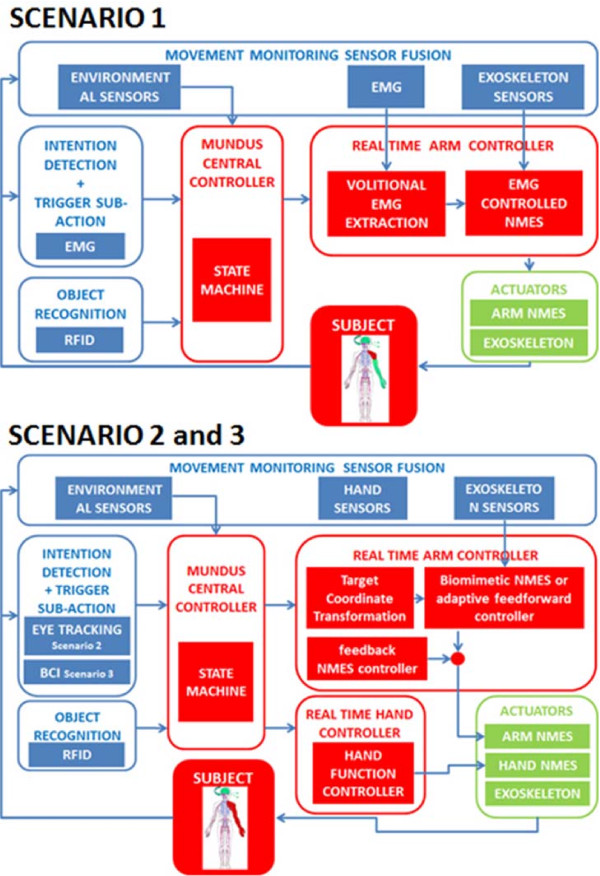
**Modules integration in the three scenarios.** Two examples of modules integration are depicted in the flowcharts corresponding to the different user scenarios. In all the flowcharts the subject block shows the condition of the user: red body districts are impaired, while green ones have still residual functional ability. The upper flowchart is referred to scenario 1. The lower flowchart is representative of both scenario 2 and 3.

MUNDUS CC activates, deactivates and controls all the non real time modules (RFID, EyeTracker, and BCI) and activates the real time controller system, that includes all other modules. The real time controller is based on a computer system running Linux/RTAI. Development and testing of the control system is performed in Scilab/Scicos environment, the realtime framework OpenRTDynamics and QRtaiLab. Figure 4 reports the integration of the MUNDUS platform in the three different scenarios.

Besides the MUNDUS CC, a graphical user interface framework – the MUNDUS GUI – has been developed with the aim to guide the caregiver through the calibration and the system initialization steps. The same GUI is used during the tasks to guide the end-user in the interaction with the system in order to tell him/her when he can activate or deactivate the brakes or trigger some of the sub-actions.

#### Power supply and safety

The MUNDUS system is powered by a 230 V main connection. To assure the safety of the system, the following safety measures have been implemented: isolating transformers for electrical safety, emergency stop button, cover for elbow brake, and warning signs to release brakes for donning/doffing.

### Performance evaluation of the MUNDUS system

Five end-users belonging to the MUNDUS target population have been recruited for the study. All the tests have been performed at the Villa Beretta Rehabilitation Centre (Valduce Hospital). The prototype and the experimental protocol for the validation of the system has been approved by the ethical committee of the Valduce Hospital and all participants signed a written informed consent. Two exemplary interaction tasks, in term of assessing the system functionality, have been selected for the first tests: drinking and reaching a body spot or a button within the working space. All the tasks were performed with the right arm since only a right-arm exo prototype has been developed. At the beginning of the session, the MUNDUS platform has been customized on the needs of each single user; thus, different configurations have been tested by different users. To assess the functionality of the system, for each subject and each performed trial, the task has been divided into sub-actions as previously described. The level of support provided by the MUNDUS system was scored for each sub-action from 0 (unsuccessful) to 1 (acceptable) and 2 (completely functional). If a sub-action was not supported by the system, a not available (NA) score was given. The scores were agreed by three experts: one was present at the tests while the other two were analysing the data and the corresponding videos.

## Results and discussions

A detailed validation of each single module on healthy subjects to completely report the fulfilment of the specifications is outside the goal of the present paper.

Five end-users with different pathologies and disability have tested the MUNDUS platform in different configurations depending on their current condition. Table 1 reports the demographic and the clinical details of the participants while Table 2 describes the MUNDUS configurations tested. In what follows, the results of the tests are described subject by subject.

**Table 1:** Characteristics of the end users

**Subject**	**Age**	**Sex**	**Pathology**	**MI Upper limb (max 0-100)**	**Fugl Meyer (max 0-44)**	**MRC right arm (max 5)**			
						**Elbow extension**	**Elbow flexion**	**Finger extension**	**Finger flexion**
**FS001**	44	M	incomplete SCI C3-C4	45	11	M3	M3	M1	M1
**RF002**	37	F	multiple sclerosis	73	29	M4	M4	M4	M4
**ND004**	79	M	incomplete SCI C4-C5	56	19	M3	M3	M2	M2
**GD007**	49	M	Multiple sclerosis	100	41	M5	M5	M5	M5
**GC008**	33	M	incomplete SCI C7-D1	23	16	M2	M2	M1	M1

**Table 2:** Configurations tested by each end-user in each session

**MUNDUS config.**	**Test**	**Scenario**	**Exo**	**Environmental sensors**	**RFID**	**Arm NMES**		**Hand**		**Intention detection**		
						**EMG controlled**	**Feedback controller**	**NMES + glove**	**Robotic orthosis**	**USB button**	**Eye tracking**	**BCI**
1	FS001test1-5	1	X	X	X							
2	FS001test6-7	1	X	X	X			X				
1	RF002test1-4	1	X	X	X							
3	ND004test1-2	2	X	X	X		X				X	
4	ND004test3	2						X				
5	GD007test1-2	1	X			X		X		X		
6	GC008test1-2	3							X			
7	GC008test3	3	X	X	X		X		X			X
8	GC008tests4-6	3	X	X			X					X

### Subject 1: FS001

**Figure 5:**
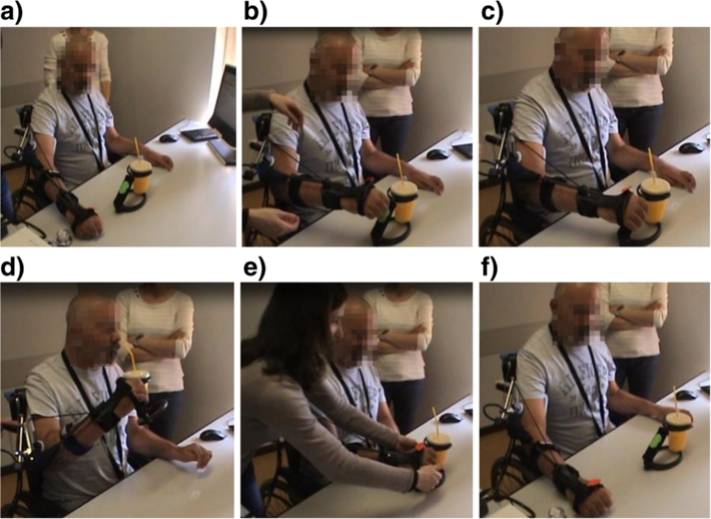
**Tests on subject FS001.** Subject FS001 movement phases during the drinking task (Additional file 2). From left to right: initial position **(a)**, reaching of the cup **(b)**, grasping of the cup **(c)**, cup to mouth **(d)**, releasing of the cup **(e)** and return to initial position **(f)**.

This subject is a quadriplegic male of 44 years old with an incomplete SCI (C3-C4 level) since 2010. This subject is classified as an ASIA Impairment Scale C with right and left motor/sensitive level C4. According to the subject’s characteristics reported in Table 1, the scenario selected was Scenario 1. To reduce the complexity of the system for the first tests, no intention detection module was used; the brakes of the exo were automatically activated once the subject reached the target position and manually de-activated by the operator when required by the subject. The subject performed two experimental sessions. In the first session he performed a drinking task exploiting only the weight compensation provided by the exo (see Figure 5 referring to-FS001_test 1 in Table 3 and Additional file 2). The subject was helped by the operator to open the hand.

**Table 3:** Evaluation of the functionality for each testing trial (NA: Not Assisted by MUNDUS; 0: unsuccessful; 1: acceptable; 2: completely functional)

**Test**	**MUNDUS config.**	**Task**	**Sub-action**									**Mean score (SD)**	**Video**
			**From rest to target**	**Open hand**	**grasp handle**	**Reach target**	**Keep position**	**Return to table**	**Release handle**	**Go back to rest**	**Intention communication**		
**FS001-test 1**	1	drinking	2	NA	NA	2	2	2	NA	2	NA	2 (0)	Additional file 2
**FS001-test 2**	1	drinking	2	NA	NA	2	2	2	NA	2	NA	2 (0)	no video available
**FS001-test 3**	1	drinking	2	NA	NA	2	2	2	NA	2	NA	2 (0)	no video available
**FS001-test 4**	1	drinking	2	NA	NA	2	2	2	NA	2	NA	2 (0)	no video available
**FS001-test 5**	1	drinking	2	NA	NA	2	2	2	NA	2	NA	2 (0)	no video available
**FS001-test 6**	2	drinking	1	1	1	2	0	2	0	2	NA	1.13 (0.83)	Additional file 3
**FS001-test 7**	2	drinking	1	1	1	2	0	2	0	2	NA	1.13 (0.83)	no video available
**RF002-test 1**	1	drinking	2	NA	NA	2	2	2	NA	2	NA	2 (0)	Additional file 4
**RF002-test 2**	1	reaching	2	--	--	2	2			2	NA	2 (0)	Additional file 5
**RF002-test 3**	1	drinking	2	NA	NA	2	2	2	NA	2	NA	2 (0)	no video available
**RF002-test 4**	1	reaching	2	--	--	2	2			2	NA	2 (0)	no video available
**ND004-test 1**	3	drinking	2	NA	NA	2	2	1	NA	1	2	1.67 (0.52)	no video available
**ND004-test 2**	3	drinking	2	NA	NA	2	2	2	NA	1	2	1.83 (0.41)	Additional file 6
**ND004-test 3**	4	drinking	NA	1.7	2	NA	2	NA	2	NA	NA	1.92 (0.15)	Additional file 7
**GD007-test 1**	5	drinking	2	2	2	2	2	2	2	2	2	2 (0)	Additional file 8
**GD007-test 2**	5	drinking	2	2	2	2	2	2	2	2	2	2 (0)	no video available
**GC008-test 1**	6	drinking	NA	2	1	NA	0	NA	2	NA	NA	1.25 (0.96)	Additional file 9
**GC008-test 2**	6	drinking	NA	2	2	NA	2	NA	1	NA	NA	1.75 (0.50)	no video available
**GC008-test 3**	7	drinking	2	2	2	1	0	0	0	0	NA	0.88 (0.99)	no video available
**GC008-test 4**	8	reaching	2	--	--	2	2			2	2	2 (0)	Additional file 10
**GC008-test 5**	8	reaching	2	--	--	2	2			2	2	2 (0)	Additional file 11
**GC008-test 6**	8	reaching	2	--	--	2	2			2	2	2 (0)	no video available
**Number of repetitions**			19	8	8	19	22	14	8	19	7		
**Mean**			1.89	1.71	1.63	1.95	1.64	1.79	1.13	1.79	2		
**SD**			0.32	0.45	0.52	0.23	0.79	0.58	0.99	0.54	0		

Only the designed support for the cup allowed the user to drink autonomously, once the operator helped him in opening the hand to grasp and then to release the handle. An extra test was done activating the brakes and repeating the task 5 times; with the exo support, the subject successfully performed the five repetitions (FS001_test 1 to 5 in Table 3); without the exo fatigue prevented the subject to repeat the task.

During the second experimental session, the subject tested the Hand NMES module to assist the opening of the hand which was not possible by his own volitional control. This session was repeated twice on two different days (FS001_test 6 and 7 in Table 3 and Additional file 3). On both days, the hand was correctly opened and closed by the stimulation. After the first day of stimulation the subject reported a positive reduction of the rigidity of the hand with the possibility to better use it to drive the wheelchair.

### Subject 2: RF002

The second subject is a female of 37 years affected by multiple sclerosis. The pathology was diagnosed in 1996. She has weakness in all of the muscles of the right arm and the pathology prevents her to perform independently activities of daily life requiring antigravity effort. According to subject’s characteristics the scenario selected was Scenario 1. Again no intention detection modules were used and the exo brakes were controlled as for the first subject. Subject RF002 was asked to perform the drinking task (RF002_test 1 in Table 3, Additional file 4) and the touching the left shoulder task (RF002_test 2 in Table 3, Additional file 5). The subject reported a perception of a more exhausting task when using the exoskeleton with respect to the natural movement. To quantitatively control whether the exo was somehow making the task execution more difficult for her, we acquired the EMG signals of the biceps and deltoids muscles. Of course this test was not intended to provide a complete evaluation of muscular fatigue but it was an evident assessment of the level of muscular activation used to perform the same task with and without the exo (RF002_test 3 and 4 in Table 3).

**Figure 6:**
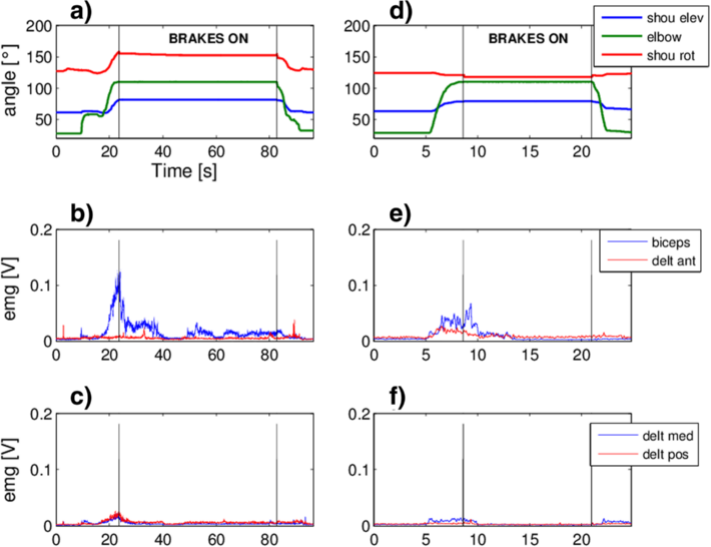
**Tests on subject RF002.** Subject RF002 angles and EMG signals measured during the drinking task **(**panels **a**-**c)** and the touching the left shoulder task **(**panels **d**-**f)**, with the support of the exo. In panels **a)** and **d)** the angles profiles are reported, the vertical lines limit the phase of the brakes activation. The correspondent EMG signals of the biceps and anterior deltoid **(**panels **b)** and **e))** and of the medial and posterior deltoid **(**panels **c)** and **f))**are reported.

Figure 6 shows the results of the drinking task (left column) and of the touching the left shoulder (right column) with the support of the exo. The breaks were activated automatically to keep the position once reached the mouth/shoulder to allow some resting to the subject and the possibility to keep the position and the function longer. The EMG activation profiles of the biceps and of the three deltoids muscles are reported in panels b), c), e) and f). It can be noticed that the subject relaxed the biceps some seconds after the activation of the brakes when she actually realized their activation.

**Figure 7:**
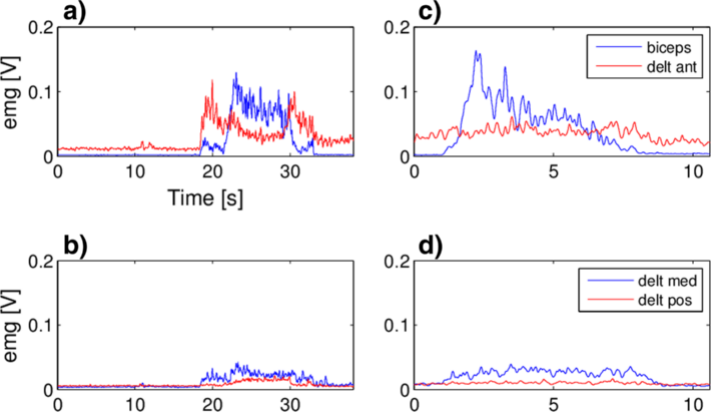
**Tests on subject RF002.** Subject RF002 EMG signals performing the drinking task **(**panels **a**-**b)** and the touching the left shoulder task **(**panels **c**-**d)** without any support. EMG signals of the biceps and anterior deltoid **(**panels **a)** and **c))** and of the medial and posterior deltoid **(**panels **b)** and **d))** are reported.

Figure 7 shows the results obtained by the same subject while performing the drinking task (left column) and the touching the left shoulder (right column) without the support of the exo. No kinematic data were available since the angle sensors are include on the exo. The subject was asked to keep the target position (the mouth or the shoulder) for at least 5 seconds.

Comparing Figure 6 and Figure 7, lower EMG activations were required when the movement was performed with the support of the exo both reducing the maximal peaks of activations, exploiting the exo antigravity support, and the duration of the activation, exploiting the brakes.

The EMG acquisitions showed that the muscles were less activated and with no evident fatiguing when supported by the exo; thus, we can conclude that, the feeling of the subject had to be partly attributed to the visual impression of the bulkiness of the exo. Anyway, her evaluation of the system was not positive, in subjective terms, i.e. acceptability and usability.

### Subject 3: ND004

This user is a quadriplegic male of 79 years old with an incomplete SCI (C4-C5 level) since 2010. This subject is classified as an ASIA Impairment Scale with right motor level C4, and left motor level C7. His residual control of the arm was very poor and he was selected to test Scenario 2 configuration. This subject carried out two different experimental sessions.

**Figure 8:**
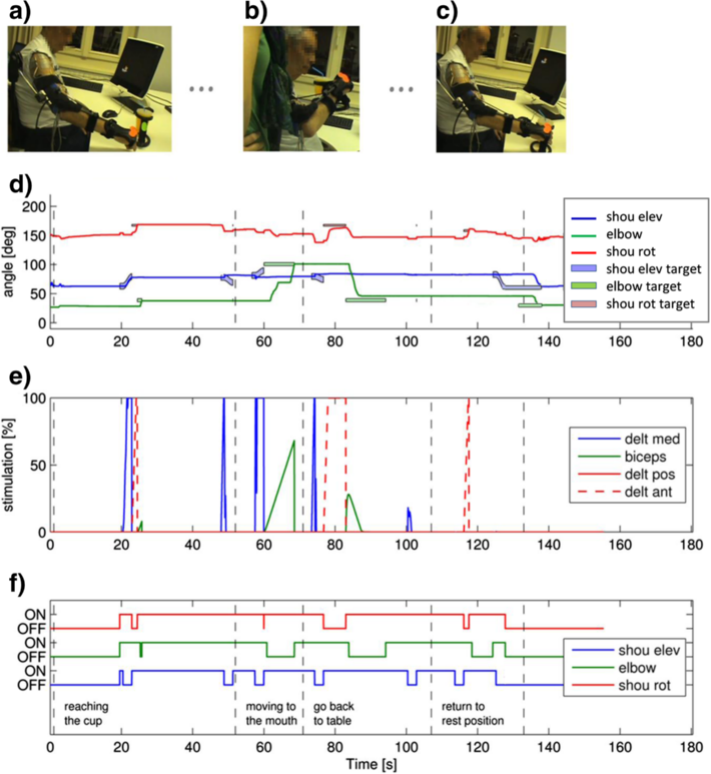
**Tests of the arm NMES on subject ND004.** Subject ND004 movement phases, stimulation and breaks activation. A complete drinking task is reported. Panels **(a**-**c)** report pictures of the subject in the initial position, at the mouth and back to rest position, respectively. In panel **d)** the angles are reported in solid lines, target angles for each phase of the task are as shadows of the same color of the correspondent angle. In panel **e)** the levels of stimulation are reported as percentage of the maximal stimulation intensity as set during the identification of the parameters on the subject. Panel **f)** reports the activation of the brakes; the indicated sentences indicate the ongoing sub-actions.

In the first session (ND004_test 1 and ND004_test 2 in Table 3. Additional file 6), the subject used scenario 2 configuration, he exploited the exo and the muscles of his right arm were stimulated with the sequential feedback control strategy to accomplish the drinking task. The subject used the eye tracking module to select the object to be grasped and to trigger the different sub-actions. The grasping and the releasing of the object were performed with the help of the operator. From MUNDUS perspective, this test aimed to testing whether the stimulation was able to assure the reaching task completion in the case of a subject with partial muscle atrophy. Figure 8 reports the results achieved during the first test performed by the subject. Pictures of the subject in three specific instants of the movement are shown: initial position (panel a), cup to mouth (panel b) and return to initial position (panel c). The figure reports also the angles profiles (panel d), the correspondent muscles stimulation (panel e) and the breaks activation (panel f) used to execute movement.

As shown in Figure 8 (panel d) the subject did reach the reference angles very nicely during the first two sub-actions, i.e. “reaching of the cup” and “moving to the mouth”, while in the second part of the task (“go back to table” and “return to rest position”) some difficulties are shown in the relaxation of the deltoids and the biceps muscles due to a residual muscles stiffness after stimulation. Indeed, these movements should have been executed thanks to gravity once the brakes were off. The persistence of some stiffness after the stimulation was observed also in some healthy subjects in the initial trials but it was soon reduced after a familiarization with the system.

Just after second 80, a sudden sliding of the shoulder horizontal rotation angle (red line in panel d) can be observed even if the correspondent brake was activated (red line in panel f). This was due to the fact the brake was not strong enough to block such a big arm. A similar problem occurred also with some healthy subjects and a new version of the horizontal shoulder brake was then integrated into the prototype. The performance of the second test improved in the second half of the drinking task a sit can be seen in the Additional file 6.

**Figure 9:**
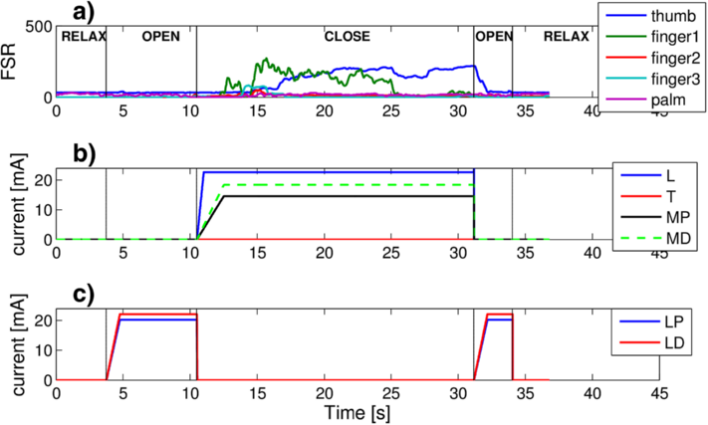
**Tests of the hand module on subject ND004.** In panel **(a)**, the force measured at the finger tips (FSR) are shown in terms of raw data having values ranging from 0 to 1023; the stimulation intensities provided to the electrodes arrays inducing the grasping and the opening of the hand are depicted in panels **b)** and **c)**, respectively.

During the second session (ND004_test 3 in Table 3, Additional file 7), Subject ND004 tested the HAND NMES module (Figure 9).

It can be noticed that when MUNDUS CC requested to open the hand, the appropriate muscles were stimulated with an increasing ramp up to the maximal tolerated current value defined in the calibration procedure (Figure 9, panel b). The opening of the thumb was not completely successful and the operator slightly helped him, however the release did not require similar assistance. On the other hand, when MUNDUS CC requested to grasp an object, stimulation pulses were delivered to the other arrays (Figure 9, panel c) and an increasing force was measured at the finger tips suggesting that an object was grasped by the subject (Figure 9, panel a).

Moreover, this subject had a lower rigidity in the hand after the stimulation session. This reduction of the stiffness allowed him to voluntarily control some opening and closing functions, otherwise not possible, also the day after the experiment.

### Subject 4: GD007

This end user is a male of 45 years. He was diagnosed with multiple sclerosis in 1988. This subject was able to perform the entire movement also without the exo support, but after the execution of few repetitions, there was a reduction of the range of motion due to a fast onset of muscular fatigue, hence he was assigned to Scenario 1. Two repetitions of the drinking task (GD007_test 1 and 2 in Table 3, Additional file 8) were performed. The subject’s arm was supported by the exo and the EMG-based NMES controller. Two muscles were stimulated according to the volitional muscular activity: the biceps and the medial deltoid. The stimulation pulse width was modulated between 0 and 450 μs according to the residual EMG activity detected by the adaptive filter [21]. The opening and closing of the hand was performed by means of the HAND NMES module. The subject preferred to use the USB button to trigger the different sub-actions because he had a good control of the left hand.

**Figure 10:**
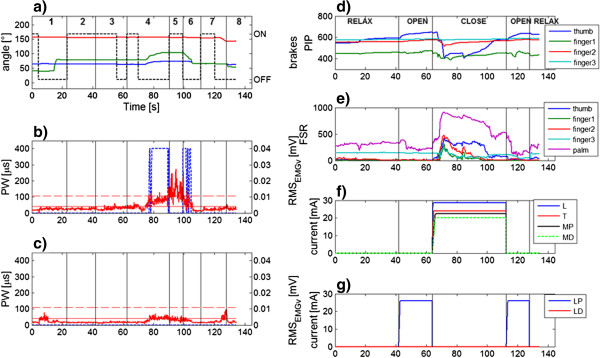
**Tests on subject GD007.** An example of the results obtained by patient GD007 during the drinking task supported by the exo, the EMG-based NMES arm module and the hand NMES module (Additional file 8) Panel **a)** shows the angles of the exoskeleton: shoulder elevation (in blue), shoulder rotation (in red), elbow angle (in green); the dashed black line shows the activation of the brakes; panels **b)** and **c)** report the root mean square of the voluntary EMG and the pulse width delivered to the muscles (the biceps and the medial deltoid are reported in panel **b)** and **c)** respectively). The activation and deactivation thresholds of the NMES controller are shown in dashed and solid horizontal line respectively. In panels **(a**-**c)** the vertical lines indicate the instants in which the subject interacted with the system and delimitate 8 different phases of the movement: 1. approach the object; 2. interaction with MUNDUS CC; 3. open hand and reach the object; 4. grasp object and move to mouth; 5. drink; 6. move back to table; 7. release object and back to rest; 8. relax hand. Data coming from the hand module are reported in the panels on the right: panel **d)** shows the kinematic raw data (range 0–1023) measured by the instrumented glove at the PIP joints, panel **e)** reports the raw data (range 0–1023) of the force sensors; the stimulation currents for the muscles involved in the grasping and hand opening are reported in panels **f)** and **g)**, respectively. In panels d-g the vertical lines indicates the different phases in terms of hand functions.

Figure 10 shows the results obtained by Subject GD007. The whole movement is divided in 8 different phases delimited by the instants in which the subject interacted with the GUI pressing the USB button (vertical lines in panel a-c). These interactions were needed to let the user decide when to activate or deactivate the brakes and when to start the hand opening and closing. Instead, the completion of the hand opening and hand closing movements were automatically recognized by the controller through the use of the sensorised glove.

The movement started with the subject reaching the object on the table (phase 1); in this phase the subject exploited only the exo and no stimulation was needed to accomplish the sub-action; once arrived close to the object, the subject decided to activate the brakes (end of phase 1). In phase 2, the system was waiting for another trigger from the user to start the opening of the hand. In phase 3, the hand was opened by NMES and the subject was getting closer to the object; when the object was reached the user triggered the grasping action (end of phase 3). Once the object was grasped by NMES, the brakes were automatically deactivated and the subject moved the cup to the mouth (phase 4). During phase 4 the arm movement was supported by NMES of only the biceps (panel b). For the medial deltoid, the support of the exo was enough to have a very small contraction to perform the task and no amplification was provided by the stimulation (panel c). Once reached the mouth (end of phase 4), the subject pushed the button in order to inform the system that the target was reached and the brakes were activated. During phase 5, the subject was drinking with all the brakes ON and the arm NMES OFF; only the hand NMES was ON to keep the grasping. Once the subject finished to drink, he pushed again the button (end of phase 5), the brakes were unlocked and the subject could move back to the table (phase 6). Once on the table, the subject triggered the hand opening (end of phase 6), the object was released and the subject went back to rest (phase 7). Finally, in phase 8 the hand was relaxed.

Concerning the hand module (Figure 10, panels d-g), the opening and closing of the hand induced by NMES was functional to grasp and release the handle of the cup.

The subject did not have any difficulty in using the system and was able to accomplish the whole task.

### Subject 5: GC008

**Figure 11:**
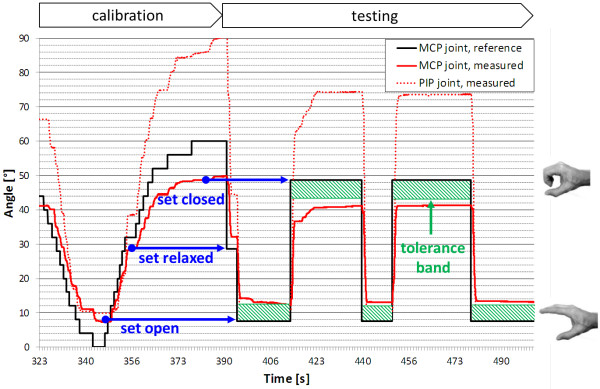
**Tests on subject GC008.** An example of the results obtained by subject GC008 while testing the robotic hand orthosis. MetaCarpoPhalangeal (MCP) and the Proximal InterPhalangeal (PIP) joint angles during the GUI-guided calibration and the subsequent testing phase are shown. The MCP joint reference is the only reference signal controlling the two coupled degrees of freedom.

This subject is a quadriplegic male of 33 years old with an incomplete SCI (C7 level) since 2011. The subject is classified as an ASIA Impairment Scale A with right and left motor/sensitive level C7. He has no residual voluntary control of his right arm and hand. Both his arm and hand muscles were completely flaccid, i.e. no muscle tone was present (see MRC scores in Table 1), and he was an NMES-responder only at the arm level. Thus, the selected scenario was Scenario 3, since the instability of his trunk control prevented the possibility to use efficiently the eye tracking module, and he tested the robotic hand orthosis. The subject carried out two experimental sessions. During the first session, the subject visited the rehabilitation centre on three consecutive days. Familiarization with the robotic orthosis, adaptation of the orthotic interface with the subject and adjustments of the orthosis as well as of the exo were the goals of the first day. On the second and third day, the subject was asked to perform two different test cases. The first test case (GC008_test 1 in Table 3, Additional file 9) involved the donning procedure of the orthosis as a stand-alone module, the GUI-guided calibration of an open, a closed and a relaxed hand position and a therapist-triggered grasp and lift movement of the drinking cup to verify the holding of the object. The grasping was not stable in this test. The same steps were performed during the second test case with the robotic orthosis mounted on the exo (GC008_test 2 in Table 3). In this second test the grasping was reliable, while the release was not completely accomplished and required the help of the operator. The arm movement for reaching the object was aided by the operator for both test cases. On the second day, the presence of the exo had no adverse effects on the performance of the tests: the cup could be securely grasped and held while the operator was moving his arm. Figure 11 shows an example of the measured MCP and PIP angles during the calibration and the subsequent grasp&hold phase. To calibrate the three hand postures, the operator incrementally increased or decreased the actuated MCP joint angle by 4° and set the values by clicking on the corresponding button on the GUI screen. The starting points of the blue arrows mark the time and angular values of these clicks. In the subsequent testing phase, the corresponding relax, open and close commands were sent to the controller. The final angles deviate from the reference angle by approximately 6° due to an implemented tolerance band and mechanical clearance. The flexible thumb brace did not always hold the thumb in a position such that it did not interfere with the cup handle. In those cases, the operator had to manually extend the thumb.

During the second experimental session, the subject tested the combination of the exo with the robotic hand orthosis and the stimulation of the arm muscles by means of the sequential feedback controller. The use of the robotic hand with the stimulation of the arm muscles (GC008_test 3 in Table 3) showed that the weight of the robotic orthosis prevented the possibility to perform the whole drinking task, since once the subject was reaching the mouth the weight of the hand system was causing a slight humeral rotation changing the orientation and preventing the correct action of the gravity to drive the return to the table sub-action. Afterwards the subject tested the following reaching tasks, without the hand module: touching the left shoulder, touching the left hand, and pushing a button (GC008_test 4 to 6 in Table 3, Additional file 10 and Additional file 11). In these last trials the Scenario 3 configuration was tested, using BCI to control the system. He was able to complete successfully all these latter tasks and to select and confirm actions by means of BCI with an accuracy of 100%.

### Performance evaluation

The evaluation scores, agreed by three experts, were assigned for each sub-action of each task performed by the subject with any support provided by the MUNDUS system and are reported in Table 3.

Overall 8 configurations of the MUNDUS system have been tested by the five end-users (Table 2). The simplest solution, including only the exoskeleton antigravity support (configuration 1) was tested by two subjects (FS001 and RF002) over 9 trials and it demonstrated a complete functionality in both the considered tasks categories, i.e. drinking and reaching. The hand NMES, as a stand-alone module (configuration 4, ND004) showed a complete functionality. When it was integrated with the rest of the system (configurations 2 and 5, FS001 and GD007 respectively) it showed some problems in the case of RF001, while a complete functionality was reported by the tests performed by GD007. Scenario 2 and 3 using the complete support of the arm functions by the sequential feedback arm NMES controller (configuration 3 and 8, ND004 and GC008 respectively) showed an overall very good performance (only for one subject there was a slight problem in the return phase because of residual stiffness). The robotic hand orthosis, tested only by GC008, had some problems in the first trial as stand-alone (configuration 6) while it had a better performance on the second one. However, its combination with the exoskeleton failed (configuration 7). The scenario 3, without hand assistance (configuration 8), was then tested over the reaching tasks and a complete functionality was assessed.

All the intention detection modules (eyetracking in configuration 3, the EMG driven in configuration 5 and the BCI in configuration 8) resulted completely reliable to permit the subjects to control the system, none of the testing subjects had problems in understanding the communication modality.

Given the complexity of the system, the preparation time including the donning, the initialization and the calibration of all the used modules is a crucial aspect to consider. The time required for the simplest configuration tested (configuration 1) ranged from a minimum of 6 minutes to a maximum of 15 minutes. Instead, when configurations including many modules are used (configuration 3 or 5) the preparation time ranged from a minimum of 35 to a maximum of 45 minutes. When also BCI is used, its calibration alone lasted about 20 minutes.

## Conclusions

MUNDUS could represent an important pioneering solution especially because of its modularity, flexibility, light and non-cumbersome features. The study is aimed at proposing the system to people at a middle stage of disability, when the effort of the individuals to restore the reduced or missed motor functions is very high. In this context, MUNDUS supports the users and follows them so to keep them as longer as possible capable to interact with their own arm in a workspace where different functions could be available.

MUNDUS contributes to improve autonomy and independence in basic activities of daily life and a better social inclusion by supplying empowerment of existing abilities and functions. Simple tasks such as drinking, scratching ourselves, changing autonomously a TV program, moving the hair away from the eyes, are among the fundamentals of our quality of life. Analogously, displacing objects or pushing buttons to start machines processes are simple works in the production line, facilitating the access to work to improve quality of life. Such tasks has been identified as the most relevant by a focus group with experts and a questionnaire gathering interviews of 36 potential users.

The pilot group of end-users have tested different configurations of the platform coping with their current level of disability. Drinking and reaching different spots either on the body or on the table have been the testing paradigms. Since these were the first tests ever performed, the system was calibrated and operated by the developers and caregivers were not involved so far. The second stage of the evaluation (currently on going) is involving also carers and therapists. Some strengths and weaknesses of the system arose from the reported experiments.

The exoskeleton well supports the weight of the arm and reduces the level of muscular activation needed to perform some daily activities. Crucially, it supports the achievement of the arm movements reducing the trunk and the head compensatory actions which are typical of impaired subjects, but which can also provoke chronic pain induced by abnormal postures. The exo can be adjusted on subjects with very different anthropometrical measures, for example consider that FS001 is a man of about 91 kg and 180 m height and RF002 is a woman of about 41 kg and 150 m height.

The use of the handle to assure the cup grasping and the independence of orientation of the object during the task has revealed as one of the most beneficial aspects for the subjects who still have a residual, but suboptimal, control of the arm and the hand.

The acceptability of the system was overall positive, only one subject was complaining about the experiments (RF002). She was a very small and weak woman not completely collaborative. During the experiment, she indeed had the impression that the exo was actually inducing an extra weight on her arm and that she was fatiguing much more in performing the tasks with the exo. EMG recordings have demonstrated that her perception was false and the exo was indeed supporting the weight of her arm during the tasks.

The arm NMES stimulation was successfully controlled in both the myo-controlled solution, scenario 1, and in the feedback controlled solution, scenario 2 and 3.

The eye tracker was effective and easy to use, as well as the BCI. The use of the eye tracker showed the advantage of a very fast and easy calibration, while BCI took longer and is more cumbersome but its use was suggested for a subject who actually had a good field of vision but a poor control of the trunk, preventing him to keep a stable posture and consequently keeping a good calibration of the eye tracker.

About the use of NMES on weak subjects, there are some limitations in the number of people who could benefit of the system because of no responsiveness to NMES, which is frequent at least in ALS people. Anyway, in the weak subjects still having residual muscular activation, the use of NMES is usually efficient, possibly after a training period to improve the functional response. Note that once the exoskeleton is supporting the weight of the arm, the muscular contractions required to accomplish the tasks are very small.

The MUNDUS system is a research prototype. An exploitation plan to transform it into a commercial device is currently ongoing by the industrial partners of the project. The complete system will not be cheap and the commercial exploitation will consider as the most likely clients the insurance companies and the health providers and not the user himself. However, one of the major advantage, currently investigated in terms of exploitation strategy, is to enlarge the possible users community at least of reduced configurations (such as the EMG controlled NMES with the exo support to be proposed for stroke survivors as upper limb rehabilitative treatment), because one of the major issue in the commercialization of the system is the low prevalence of the target pathologies.

## Abbreviations

MUNDUS: MUltimodal Neuroprosthesis for Daily Upper limb Support; ICF: International Classification of Functioning, Disability and Health; Exo: Exoskeleton; SCI: Spinal cord injury; ALS: Amyotrophic Lateral Sclerosis; EMG: ElectroMyoGraphy; EEG: ElectroEncephaloGraphy; NMES: Neuro Muscular Electrical Stimulation; RFID: Radio Frequency Identification; BCI: Brain Computer Interface; ERP: Event Related Potentials; FSR: Force Sensing Resistors; DOF: Degrees Of Freedom; MUNDUS CC: MUNDUS Central Controller; ROM: Range Of Motion; MD: Medial Distal; MP: Medial proximal; LD: Lateral Distal; LP: Lateral Proximal; P: Palmar; T: Thenars; MCP: MetaCarpoPhalangeal joint angle; MS: Multiple Sclerosis; PIP: Proximal InterPhalangeal joint angle; NA: Not available.

## Competing interests

The authors have no competing interests in relation to this study.

## Authors’ contributions

AP participated to conception, coordination and implementation of the whole research project, set-up integration and data collection, manuscript writing. SF, EA participated to implementation of the whole research project and set-up integration, NMES-module design and development, data collection, manuscript writing. MG_it, CC participated to the eye-tracking module design, development and integration. TS, CK participated to the arm controller strategies design, development and integration. JP, CV participated to the BCI-module design, development and integration. MG_at, WR, JK participated to the exo design, development and integration. SM, AC participated to the hand-module design, development and integration. FM, MR, GP, EG participated to the requirement definition, patients recruitment, data collection and dealt with the clinical issues. AJ, MH participated to the environmental sensors design, development and integration. MB, EDA participated to the RFID design, development and integration. PS, SZ, ADW, JM, LG participated to the HW and SW integration of all modules. GF participated to conception, coordination and implementation of the whole research project. All authors contributed to manuscript review. All authors read and approved the final manuscript.

## Supplementary Material

Additional file 1 **Documentation: Focus group and user group questionnaires.** The document reports the questions used to drive the focus group work and the corresponding results and the questionnaire of the potential user group along with a summary of the answers. Click here for file

Additional file 2 **The movie shows the end-user FS001 performing the drinking task using the MUNDUS system.** The following modules are used exoskeleton for weight support; environmental sensors for detecting object position RFID to identify the object. Click here for file

Additional file 3 **The movie shows the end-user FS001 performing the drinking task using the MUNDUS system.** The following modules are used: passive exoskeleton for weight support; environmental sensors for detecting object position; RFID to identify the object; hand NMES to perform the grasping; sensorised glove to measure the kinematics of the fingers and the stability of the grip. Click here for file

Additional file 4 **The movie shows the end-user RF002 performing the drinking task using the MUNDUS system.** The following modules are used: exoskeleton for weight support; environmental sensors for detecting object position; RFID to identify the object. Click here for file

Additional file 5 **The movie shows the end-user RF002 performing the reaching task toward the shoulder using the MUNDUS system.** The following modules are used: exoskeleton for weight support; environmental sensors for detecting object position; RFID to identify the object. Click here for file

Additional file 6 **The movie shows the end-user ND004 performing the drinking task using the MUNDUS system.** The hand was supported by the operator during the task. The following modules are used: exoskeleton for weight support; environmental sensors for detecting object position; RFID to identify the object; arm NMES for performing the reaching movements (feedback controller); eye tracking for intention detection and triggering of the sub-actions. Click here for file

Additional file 7 **The movie shows the end-user ND004 testing the HAND NMES as a stand alone module.** The following modules are used: hand NMES to perform the grasping; sensorised glove to measure the kinematics of the fingers and the stability of the grip. Click here for file

Additional file 8 **The movie shows the end-user GD007 performing the drinking task using the MUNDUS system.** The following modules are used: exoskeleton for weight support; arm NMES to support the reaching task (EMG based NMES controller); hand NMES to perform the grasping; sensorised glove to measure the kinematics of the fingers and the stability of the grip; USB button for intention detection and triggering of the sub-actions. Click here for file

Additional file 9 **The movie shows the end-user GC008 testing the robotic hand orthosis as a stand-alone module.** The following modules are used: the robotic hand orthosis to provide hand grasping and releasing functions. Click here for file

Additional file 10 **The movie shows the end-user GC008 performing the reaching the button task using the MUNDUS system.** The following modules are used: exoskeleton for weight support; environmental sensors for detecting object position; arm NMES to perform the reaching task (feedback controller); brain computer interface for intention detection and triggering of the sub-actions. Click here for file

Additional file 11 **The movie shows the end-user GC008 performing the reaching the shoulder task using the MUNDUS system.** The following modules are used: exoskeleton for weight support; environmental sensors for detecting object position; arm NMES to perform the reaching task (feedback controller); brain computer interface for intention detection and triggering of the sub-actions. Click here for file
