# Supplementary material for: MUNDUS project: MUltimodal Neuroprosthesis for daily Upper limb Support
Source: J Neuroeng Rehabil. 2013 Jul 3;10:66. doi: 10.1186/1743-0003-10-66 (PMC3733825; doi:10.1186/1743-0003-10-66)
Supplement: Additional file 1 — Documentation: Focus group and user group questionnaires. The document reports the questions used to drive the focus group work and the corresponding results and the questionnaire of the potential user group along with a summary of the answers. [file 1743-0003-10-66-S1.pdf]

1.1.1. FOCUS GROUP QUESTIONS

Eight questions were submitted to the participants:

Q1. For a subject with some residual control of the muscles of the arm and hand: which may be the primary needs that the planned device could fulfill? Could you give a priority order?

Q2. For a subject without any residual muscles activity of the upper limb but with some residual movement of head and eyes: which may be the primary needs that the planned device could fulfill? Could you give a priority order?

Q3. For a subject without no residual control of any muscles: which may be the primary needs that the planned device could fulfill? Could you give a priority order?

Q4. In your opinion where would this device by more useful? At home? At work? Both? Other?

Q5. In your opinion which could be the expectations of a potential user about this type of device?

Q6. In your opinion which are the features the device must have in order to be usable during the evolution of the specific pathology?

Q7. In your opinion which are the main movements that this device should favour (elbow flex/ext, shoulder abd/add, etc)?

Q8. In your opinion the device should be wearable or it may be fixed to a support?

Each participant gave his/her answer for each question.

1.1.2. Main Results

For each question the main results are:

Q1. For the scenario indicated in this question the higher priority activities are: activation of alarm system, call for something, autonomous eating, drinking and personal care, helping the other hand, touch own body (scratch oneself), help residual function for working goals.
The associated movements are: anterior reaching, pushing/pooling, grasping/release, hand to mouth movement, hand to body movement, fingers movement.

Q2. For the scenario indicated in this question the higher priority activities are: activation of alarm system, call for something, support for eating, drinking and personal care,
The associated movements are: anterior reaching, pushing/pooling, grasping/release, hand to mouth movement.

Q3. Some difficulties were observed to identify the specific needs under these scenario. The higher priority activities are: little movement of some body segments for postural needs, head motion and control, call for help, push a button, communications.
The associated movements are: hand to body movement, head movement and control, reaching and push.

Q4. Participants have not indicated specific places. They have just suggested some general trends, related to the development of each pathology. For instance, for the first scenario it may be useful at home and at work, for the second scenario

it could be useful at home and in some cases for social participation, and in general for the third scenario it may be useful just at home and if possible wearable.

Q5. Participants have distinguished the expectations based on the different scenarios.
Scenario 1: fatigue reduction, higher speed and precision of movements, facilitation of rehabilitation, mastering the situation, compensation, to be useful in society, the solutions of all problems, increasing/maintaining autonomy, recover some movement abilities like interaction with the body, handling and grasping. Scenario 2: performance of volunteer actions, performance of basic functions, recover lost functions, solution to a good deal of problems, increasing autonomy. Scenario 3: reduction of pain and discomfort, change own body position, be able to do some movement allowing interaction, not to feel alone, change a difficult situation, a little more autonomous, personal dignity and survival.

Q6. Participants have identified few but clue features: modularity, costs, transportability, easy to use/simple, easy passages during the evolution of the pathology (avoiding re-training), efficacy, to keep the same interface and working in different phases, wearable, biomimetic control, not heavy, do not force the patient.

Q7. The participants indicated following movements: Elbow flexion/extension, Shoulder abduction/adduction, Whole upper limb movement, Combined motion shoulder/elbow and wrist support incoming/outgoing
It seems that all movements are equally important for participants. On second place they have mentioned the finger grasping movement.

Q8. Most of participants think the device should be wearable, but some of them including the patient, think that it may be even wearable or fixed, depending on the specific condition.

1.1.3. POTENTIAL USER GROUP QUESTIONNAIRE

Six questions with the correlate options had been submitted to the subject:

Q1. If we tell you that there is a device like the one of the project: what do you expect it will be permit you to do?

- A1. Move arm better
- A2. Just move arm
- A3. Eat
- A4. Drink
- A5. Personal Hygiene
- A6. Help other arm
- A7. Have more autonomy
- A8. Perform some activity
- A9. Rest the arm
- A10. Push a button
- A11. Use PC
- A12. Other (specify)

Q2. Where would you prefer to use this device the most?

At Home

- A1. Bathroom
- A2. Kitchen
- A3. Bedroom

A4. Living room
A5. Home garden

At Work/school

A6. perform specific tasks
A7. help other arm
A8. work in general

Other (specify)

Q3. In which moment of the day do you think the device would be more useful?
A1. Morning
A2. Midday
A3. Afternoon
A4. Evening
A5. Always
A6. Other (specify)

Q4. Why do you think that in the period indicated in question 3 it would be more useful?
A1. Free text answer

Q5. In your opinion, which should be the most important characteristics of this device?
A1. Light
A2. Easy to use
A3. Wearable
A4. Fixed on a support
A5. Other (specify)

Q6. Which kind of movement do you think are more useful for you?
A1. arm up/down
A2. hand to body
A3. Grasping
A4. hand to mouth
A5. Other (specify)

Each participant could choice one or more answer for each question.

1.2. Results of User group feedback

1.2.1. Main Results

For each question the main results are:

Q1. Most of the answers reflect a need of increasing autonomy mainly related to specific activities of daily living (ADL):

- eat
- drink
- personal hygiene
- When the choice is "other" the content is:
  - Increase the possibility to go outdoors
  - Dressing
  - Control wheelchair
  - Reaching in general

Q2. Most of these patients foresee activities mainly at home. The preference for Kitchen and Bathroom as main places for using the device is consistent with answers given to question 1

- When the choice is "other" the content is:
  - Going outdoors
  - Driving
  - Going to University
  - Help the other arm (in any context)

Q3. There is a clear preference to use it always.

- When the choice is "other" the content is:
  - Meal hours

Q4. The answers correspond to two main criteria:

- Relationship with the ADL they have previously indicated
- The moment of the day they are not usually at bed

Q5. The preference seems to point to a device:

- Light
- Wearable
- Easy to use
- When the choice is "other" the content is:
  - Biomimetic

Q6. Answers to this question are also consistent with the ADL previously indicated, that is a preference for movement:

- Grasping
- Arm Up/Down
- Hand to mouth
- When the choice is "other" the content is:
  - Arm extension
  - Finger movement (using keyboard)
  - Pushing (wheelchair)
